# Triple Blockade of Oncogenic RAS Signaling Using KRAS and MEK Inhibitors in Combination with Irradiation in Pancreatic Cancer

**DOI:** 10.3390/ijms25116249

**Published:** 2024-06-06

**Authors:** Xuan Wang, Johanna Breuer, Stephan Garbe, Frank Giordano, Peter Brossart, Georg Feldmann, Savita Bisht

**Affiliations:** 1 Department of Internal Medicine 3, Center of Integrated Oncology (CIO-ABCD) Aachen-Bonn-Cologne-Düsseldorf, University Hospital of Bonn, Venusberg Campus-1, 53127 Bonn, Germany; 2 Institute of Molecular Medicine and Experimental Immunology, University Hospital of Bonn, Venusberg Campus-1, 53127 Bonn, Germany; 3 Department of Radiology and Radiation Oncology, University Hospital of Bonn, Venusberg Campus-1, 53127 Bonn, Germany

**Keywords:** pancreatic cancer, KRAS inhibitors, MEK inhibitor, irradiation, combination therapy, RAS/MAPK pathway

## Abstract

Pancreatic ductal adenocarcinoma (PDAC) is one of the deadliest of human malignancies and carries an exceptionally poor prognosis. It is mostly driven by multiple oncogenic alterations, with the highest mutation frequency being observed in the KRAS gene, which is a key oncogenic driver of tumorogenesis and malignant progression in PDAC. However, KRAS remained undruggable for decades until the emergence of G12C mutation specific KRAS inhibitors. Despite this development, this therapeutic approach to target KRAS directly is not routinely used for PDAC patients, with the reasons being the rare presence of G12C mutation in PDAC with only 1–2% of occurring cases, modest therapeutic efficacy, activation of compensatory pathways leading to cell resistance, and absence of effective KRASG12D or pan-KRAS inhibitors. Additionally, indirect approaches to targeting KRAS through upstream and downstream regulators or effectors were also found to be either ineffective or known to cause major toxicities. For this reason, new and more effective treatment strategies that combine different therapeutic modalities aiming at achieving synergism and minimizing intrinsic or adaptive resistance mechanisms are required. In the current work presented here, pancreatic cancer cell lines with oncogenic KRAS G12C, G12D, or wild-type KRAS were treated with specific KRAS or SOS1/2 inhibitors, and therapeutic synergisms with concomitant MEK inhibition and irradiation were systematically evaluated by means of cell viability, 2D-clonogenic, 3D-anchorage independent soft agar, and bioluminescent ATP assays. Underlying pathophysiological mechanisms were examined by using Western blot analyses, apoptosis assay, and RAS activation assay.

## 1. Introduction

Pancreatic ductal adenocarcinoma (PDAC) is one of the deadliest cancers and the seventh leading cause of cancer-related death globally [1]. In the United States and Europe, it is the third leading cause of cancer-related death and is projected to become the second leading cause before 2040 [2,3]. Despite significant advances in surgery and multi-agent chemotherapy, PDAC remains recalcitrant, with a gloomy 5-year survival rate of <10% that has not changed appreciably over the last four decades [4,5]. The only potentially curative therapeutic option for PDAC consists of surgical resection of localized tumors, which is achievable in less than 20% of all cases. But even in these cases, long-term survival is rare, and most patients die within 10–20 months after surgery. The factors attributing to the lethality of PDAC are numerous and include lack of early symptoms or late diagnosis at advanced stage, strong resistance to chemotherapy, and ineffective adjuvant chemotherapy [6]. Therefore, there is a great need for effective therapies where the major focus has been to identify therapeutically actionable genomic alterations in PDAC patients, who might benefit from targeted therapies matched to their molecular profile [7]. Genetically, PDAC progresses as a result of the activation of oncogenes and inactivation of tumor suppressors, with RAS oncogene being the most predominant one amongst all [8,9]. PDAC is characterized by a high mutation rate (up to 95%) in the Kirsten rat sarcoma virus oncogene homolog (KRAS) gene, and a few cases with wild-type (wt) KRAS that usually harbor mutations in BRAF [10,11,12]. Activating KRAS mutations are among the earliest oncogenic genomic alterations found in pancreatic intraepithelial neoplasia (PanIN) precursor lesions and seem to be a prerequisite for the development of a fully invasive metastatic pancreatic cancer phenotype [13,14,15]. At the same time, PDAC cells appear to maintain dependence on oncogenic Ras signaling for survival and proliferation [16,17,18]. Oncogenic KRAS variants signal through various well-described carcinogenic signaling pathways, including RAF-mitogen-activated protein kinase (RAF-MAPK), phosphoinsositide-3-kinase (PI3K), and Ral GDS pathways. An overview of KRAS downstream effector pathways is provided [10]. Therefore, pharmacologically inhibiting oncogenic KRAS signaling has been suggested for a long time and is still considered as the “Holy Grail” in the quest for targeted therapeutic approaches directed against pancreatic cancer by many authors [19]. However, a major obstacle has been that this most frequently observed molecular alteration in PDAC remained an intractable therapeutic target for decades until the emergence of first efficacy data of KRAS G12C inhibitors that increased optimism about next-generation KRAS-directed therapies in PDAC [11,12,20]. Most existing KRAS G12C inhibitors such as Sotorasib or MRTX849 have shown remarkable efficacy in KRAS G12C driven cancers such as non-small cell lung cancer [21,22,23,24] but limited effect on PDAC due to the rare occurrence of G12C mutation cases. To date, no suitable inhibitors directly targeting G12D/G12V, which forms the majority of KRAS mutations in PDAC, are available for routine application outside of early clinical and preclinical studies [23]. Therapeutic approaches targeting KRAS mutation indirectly by inhibiting downstream effectors such as MEK1/2, ERK1/2, or AKT were also attempted [25,26,27,28,29] but only few are found to be truly effective in suppressing KRAS mutation and related pathways in PDAC [30,31]. Moreover, most clinical trials focused on inhibiting downstream effector pathways showed modest or no clinical benefit, the reasons being the small therapeutic window causing toxicity and activated compensatory pathways leading to chemo-resistance [32].

Also, accumulating preclinical evidence already suggests that, though it is a potentially powerful therapeutic approach, pharmacological inhibition of oncogenic KRAS signaling alone will not likely be sufficient to fully eradicate and cure metastatic pancreatic cancer [17,19]. Therefore, identification of effective combinatorial strategies that induce synthetic lethality with KRAS inhibition at various points along the same oncogenic pathway is crucial for minimizing resistance and underscores the significance of understanding feedback signaling mechanisms involving upstream receptor tyrosine kinases such as Epidermal Growth Factor Receptor (EGFR), which may act as an upstream mediator of oncogenic KRAS signaling and therefore represents a potential target for therapeutic intervention, KRAS, and its downstream effector pathways during the evaluation of different KRAS inhibitors. A study carried out using this combination approach has recently shown promising potential to enhance therapeutic efficacy [33].

Additionally, some clinical data also linked aberrant oncogenic KRAS signaling to radiotherapy resistance in tumors [34,35]. Numerous lines of other evidence have also pointed out hyperactivation of RAS/MAPK pathway leading to the development of radioresistance [36,37,38]. Although, the role of radiation therapy in pancreatic cancer is not well established and is still being explored as a component of the standard treatment regimen [39,40], so far pancreatic cancer has been proven to be difficult to treat by radiotherapy [41]. So, molecularly targeted therapies that enhance the effectiveness of radiation might be an attractive option for treating pancreatic cancer. Therefore, the purpose of this study is to test the hypothesis that KRAS/MEK inhibitors radiosensitize RAS-driven malignancies and might offer clear therapeutic benefit when integrated with radiotherapy. Small molecule inhibitors of KRAS, SOS1/2, or MEK were subsequently evaluated for their radiosensitizing potential alone or in combination.

## 2. Results

### 2.1. Combination Treatment with KRAS- and MEK-Inhibitor Plus Irradiation Inhibits Cell Growth of KRAS Mutant Pancreatic Cancer Cells

Viability of pancreatic cancer cell lines with different KRAS mutational status, namely MiaPaCa (KRAS G12C), Panc-1 (KRAS G12D), and BxPC3 (KRAS wild-type), was analyzed using Celltiter-Glo luminescent assay, which relies on the quantitation of ATP present in the cells as the measure of metabolically active cells. In this assay, the efficacy of triple combination treatment with KRAS-inhibitors, MEK-inhibitor, and low-dose irradiation (i.e., KRASi + MEKi + IR) was tested and compared with double combination therapy, i.e., combination of two small molecule inhibitors (KRASi + MEKi) without concomitant irradiation. Additional combination regimens (KRASi + IR and MEKi + IR) were also compared to each monotherapy individually (KRASi, MEKi, or IR monotherapy) as well as to mock controls.

**Figure 1:**
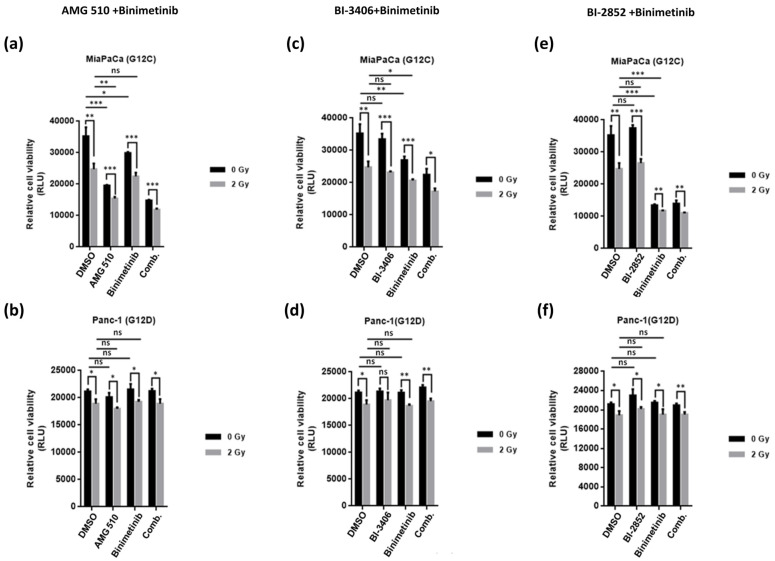
Relative cell viability of MiaPaca**^KrasG12C^** and Panc-1**^KrasG12D^** cells treated with AMG-510 (**a**,**b**), BI-3406 (**c**,**d**), and BI-2852 (**e**,**f**) at a concentration of 10 nM, 100 nM, and 1 µM, respectively, either singly or in combination with Binimetinib used at a concentration of 10 nM exposed to either low-dose irradiation (2 Gy) or no irradiation (0 Gy). The mitochondrial metabolic function (viability) is plotted as relative luminescence unit (RLU) of treated cells in relation to mock treated cells. Results of three independent experiments are shown and are expressed as means ± SD. Statistical significance has been indicated as * *p* < 0.05, ** *p* < 0.01, *** *p* < 0.001, ns = non-significant.

As shown in Figure 1, three different inhibitors targeting KRAS directly or indirectly (AMG510, BI-3406, or BI-2852) were used either alone or in combination with the MEK inhibitor Binimetinib and exposed to low-dose irradiation of 2 Gy or no irradiation (0 Gy), respectively. Interestingly, cell viability for either MiaPaCa or Panc-1 cell lines was markedly reduced by irradiation alone. However, when irradiation was combined with KRAS- and MEK-inhibition, the combined effect was found to be more pronounced in either cell line as compared to the effect of the KRAS-MEK combination without irradiation. BxPC-3 cells with wild-type KRAS did not show any tangible effect in terms of growth reductions upon the combined KRAS-MEK-inhibition at these concentrations, with or without concomitant irradiation (Supplementary Figure S1).

As shown in Figure 1a,c,e MiaPaCa cells were found to be more sensitive to AMG-510+Binimetinib, BI-3406+Binimetinib, or BI-2852+Binimetinib combinations, respectively, as compared to Panc-1 cells, as shown in Figure 1b,d,f. When these drug combinations were exposed to low-dose radiation of 2 Gy, decrease in cell viability was significantly enhanced as compared to the respective drug combinations without irradiation.

Moreover, among the three inhibitors targeting KRAS, AMG-510 monotherapy at a dose of 10 nM led to marked growth inhibition of KRAS G12C mutant MiaPaCa cells, while KRAS G12D mutant Panc-1 cells were not affected, in line with G12C allele-specificity of this compound. As opposed to these findings, neither BI-3406 nor BI-2852 monotherapy applied at their respective concentrations of 100 nM or 1 µM, respectively, showed any tangible in vitro therapeutic efficacy in either cell line. However, low-dose radiation alone showed significant cell growth inhibition in MiaPaCa cells or Panc-1 cells, and there was no therapeutic synergism with either BI-3406 or BI-2852, given alone or in combination with Binimetinib in this setup.

### 2.2. Combination Treatment Using KRAS- and MEK-Inhibitors Radiosensitizes KRAS Mutant PDAC Cells and Inhibits Clonogenicity

**Figure 2:**
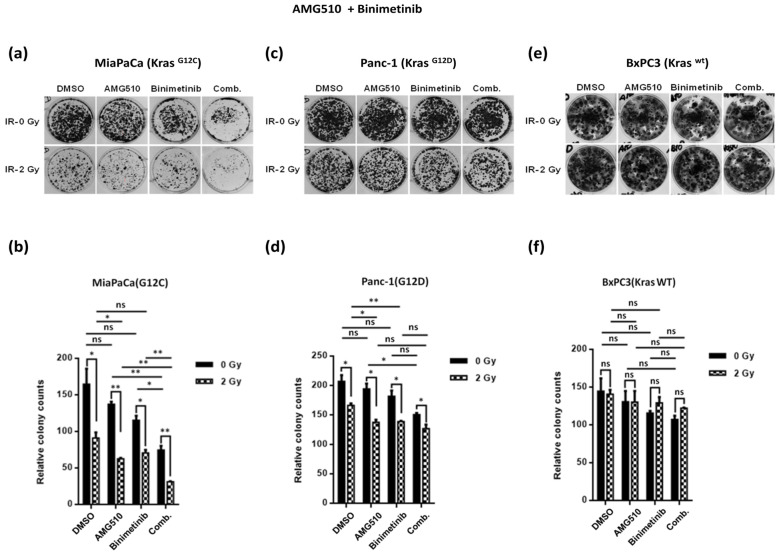
Representative images of colony formation assay for MiaPaCa**^KrasG12C^**, Panc-1**^KrasG12D^**, and BxPC3**^KrasWT^** cells treated with AMG-510 at a concentration of 10 nM and Binimetinib at a concentration of 10 nM either singly or in combination with each other in the presence of low-dose irradiation (2 Gy) or no irradiation (0 Gy) (**a**,**c**,**e**). Colonies formed were counted and plotted, where the drug-treated cells were normalized to DMSO-treated cells in their respective irradiation group (**b**,**d**,**f**). The results from three independent experiments are shown and expressed as means ± SD, and the level of significance has been indicated accordingly (* *p* < 0.05, ** *p* < 0.01, ns = non-significant).

To assess whether KRAS and MEK inhibitor combinations can induce radiosensitivity in KRAS mutant or KRAS wildtype cancer cells, two-dimensional clonogenic assays were performed. In line with results obtained using growth inhibition assays, dual combination therapy of AMG-510 plus Binimetinib significantly abrogated colony formation of G12C mutant MiaPaCa cells, and this effect was more pronounced when the cell line was exposed to low-dose radiation of 2 Gy. These data thus hint at a potential therapeutic synergism of combined KRAS and MEK inhibition by AMG510 plus Binimetinib in combination with 2 Gy irradiation in KRAS G12C mutated MiaPaCa pancreatic cancer cells (Figure 2a,b). As expected, AMG510 and Binimetinib when used either as dual combination or monotherapy did not confer any synergism or additive effect in the G12D mutated cell line but showed significant reduction in the number of colonies formed only when exposed to irradiation (Figure 2c,d). In line with the supposed mechanisms of action, neither KRAS inhibition by means of AMG510 nor MEK inhibition with Binimetinib showed any tangible reduction in clonogenicity on KRAS wild type BxPC3 pancreatic cancer cells, and there was no additive or synergistic effect upon combination with irradiation (Figure 2e,f).

**Figure 3:**
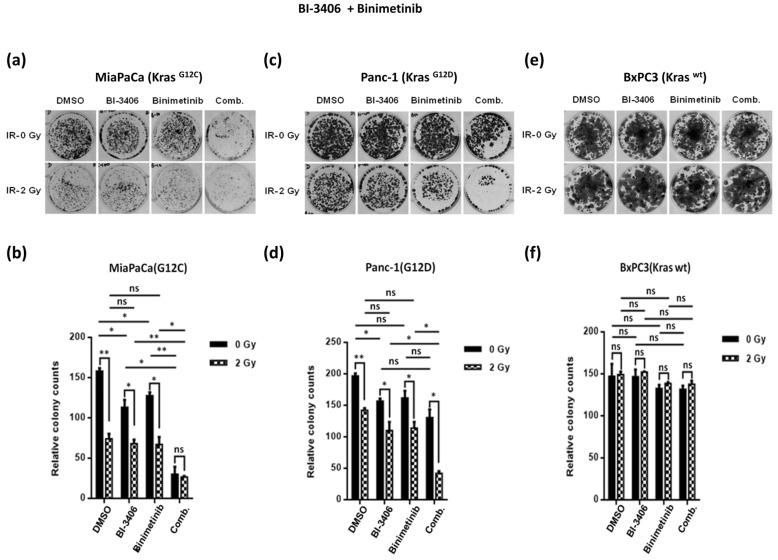
Representative images of colony formation assay for MiaPaCa**^KrasG12C^**, Panc-1**^KrasG12D^**, and BxPC3**^KrasWT^** cells treated with BI-3406 at a concentration of 100 nM and Binimetinib at a concentration of 10 nM either singly or in combination with each other in the presence of low-dose irradiation (2 Gy) or no irradiation (0 Gy) (**a**,**c**,**e**). Colonies formed were counted and plotted, where the drug-treated cells were normalized to mock treated cells in their respective irradiation group (**b**,**d**,**f**). The results from three independent experiments are shown and expressed as means ± SD, and the level of significance has been indicated accordingly (* *p* < 0.05, ** *p* < 0.01, ns = non-significant).

BI-3406, on the other hand, showed moderate therapeutic in vitro efficacy in both KRAS G12C and G12D mutated pancreatic cancer cell lines, MiaPaCa and Panc-1, respectively, in terms of reduction of clonogenicity, which was slightly more pronounced upon combination therapy with BI-3406 plus Binimetinib. Of note, however, additional irradiation was found to confer pronounced additional reduction in clonogenicity in both of the KRAS mutant cancer cell lines tested, suggesting potential therapeutic efficacy of KRAS inhibition by BI-3406 in combination with MEK inhibition and irradiation (Figure 3a–d). Again, on-target efficacy was suggested by the apparent lack of any therapeutic efficacy of BI-3406, Binimetinib, or radiation therapy as well as combination regimens in KRAS wild type BxPC3 pancreatic cancer cells (Figure 3e,f).

**Figure 4:**
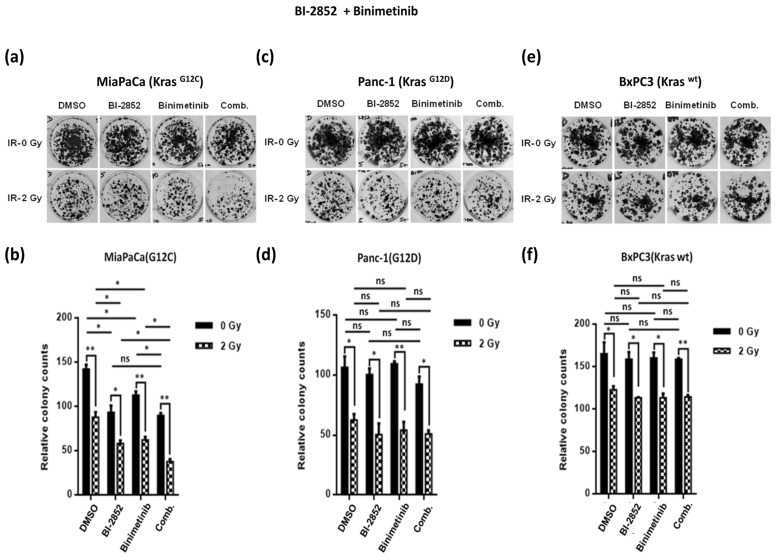
Representative images of colony formation assay for MiaPaCa**^KrasG12C^**, Panc-1**^KrasG12D^**, and BxPC3**^KrasWT^** cells treated with BI-2852 at a concentration of 5 µM and Binimetinib at a concentration of 10 nM either singly or in combination with each other in the presence of low dose irradiation (2 Gy) or no irradiation (0 Gy) (**a**,**c**,**e**). Colonies formed were counted and plotted, where the drug treated cells were normalized to mock treated cells in their respective irradiation group (**b**,**d**,**f**). The results from three independent experiments are shown and expressed as means ± SD and the level of significance has been indicated accordingly (* *p* < 0.05, ** *p* < 0.01, ns = non-significant).

The third KRAS inhibitory compound tested here, BI-2852, that acts as a pan-KRAS inhibitor for the switch I/II pocket, showed moderate reduction of clonogenicity in KRAS G12C mutant MiaPaCa cells with additive reduction upon combined irradiation (Figure 4a,b), but there was no tangible efficacy in the KRAS G12D mutant Panc-1 or KRAS wt BxPC3 cells, respectively, suggesting this compound is a less promising drug candidate for pancreatic cancer therapy at least in this specific experimental setup applied here (Figure 4c–f).

### 2.3. Combined KRAS and MEK Inhibition plus Irradiation Leads to Enhanced Attenuation of 3D Anchorage Independent Colony Growth in KRAS Mutant PDAC Cells

To further examine the combined therapeutic efficacy of KRAS and MEK inhibitors on radiosensitization, three-dimensional anchorage independent soft agar colony formation assays were performed on KRAS mutant cell lines after exposing them to irradiation alone or in combination with KRAS- and MEK-inhibitors.

**Figure 5:**
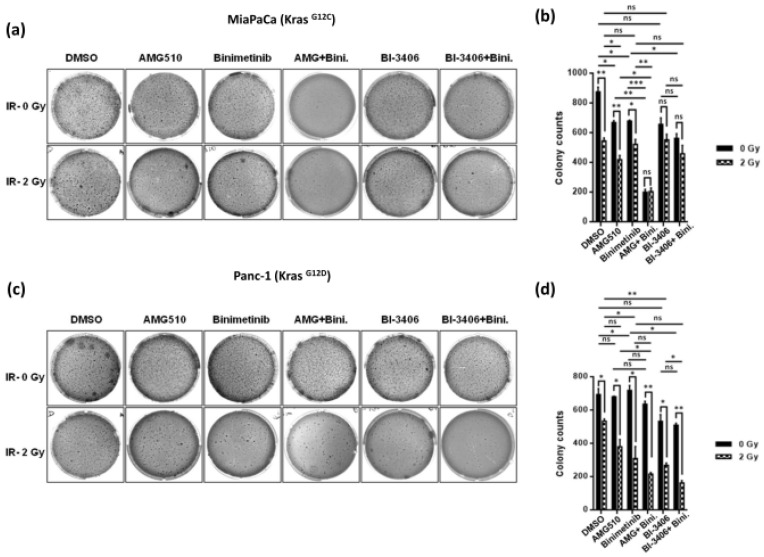
Representative images illustrating anchorage independent growth of cell colonies in three dimensional (3D) matrix for MiaPaCa**^KrasG12C^** and Panc-1**^KrasG12D^** cells treated with either AMG-510 at a concentration of 10 nM or BI-3406 at a concentration of 100 nM in combination to Binimetinib used at a concentration of 10 nM in the presence of low-dose irradiation (IR-2 Gy) or no irradiation (IR- 0 Gy) (**a**,**c**). Colonies formed were counted and plotted, where the drug-treated cells were normalized to mock treated cells in their respective irradiation group (**b**,**d**). Data represented as means ± SD for three independent experiments, and statistical significance was stated accordingly (* *p* < 0.05, ** *p* < 0.01, *** *p* < 0.001, ns = non-significant).

As shown in Figure 5a, both AMG-510 and BI-3406 significantly reduced colony formation and anchorage independent growth of KRAS G12C mutant MiaPaCa pancreatic cancer cells. For AMG510, this effect was more pronounced upon combination with either MEK inhibition by means of Binimetinib treatment or concomitant irradiation, while there was no apparent additional benefit of triple therapy of AMG510 plus Binimetinib treatment plus irradiation at these particular experimental conditions, likely due to near-complete abrogation of colony growth through combined KRAS and MEK inhibition with AMG510 plus Binimetinib combination treatment alone (Figure 5a,b).

There was a numerical reduction in colony counts upon combined KRAS inhibition with BI-3406 plus Binimetinib treatment in combination with irradiation, but this reduction did not reach statistical significance when applied in KRAS G12C mutant MiaPaCa cells (Figure 5a,b).

In KRAS G12D mutant Panc-1 pancreatic cancer cells, neither AMG510 nor Binimetinib treatment alone caused significant reduction of colony growth in soft agar assays, while there was some degree of additive reduction in colony counts upon combined MEK inhibition with irradiation. Of note, however, in this model there was marked therapeutic efficacy of BI-3406 monotherapy in terms of reduced colony growth as compared to the mock treatment. Moreover, this effect was significantly enhanced upon combinatorial KRAS inhibition by means of BI-3406 treatment plus MEK inhibition with Binimetinib and irradiation (Figure 5c,d). These observations suggest this combination as a potentially promising approach to target KRAS G12D mutant pancreatic cancer cells.

### 2.4. KRAS Inhibitors AMG510 and BI-3406 Inhibit KRAS Activation and Signaling through the RAS/MAPK Pathway in KRAS Mutant Pancreatic Cancer Cells

**Figure 6:**
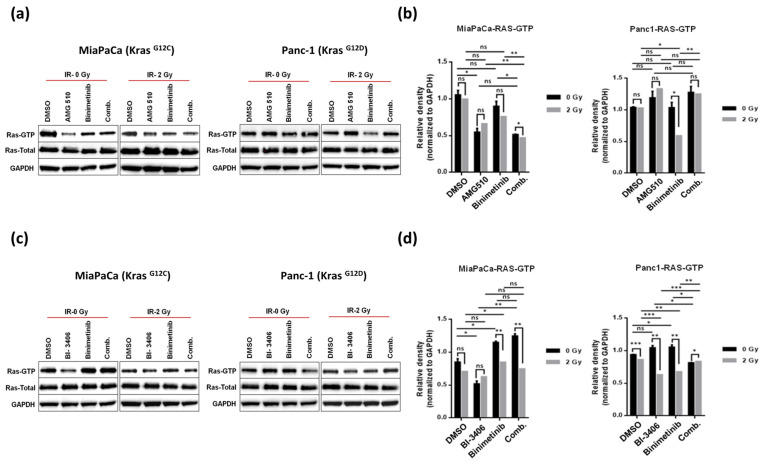
Pull-down assay of activated RAS performed on MiaPaCa**^KrasG12C^** and Panc-1**^KrasG12D^** treated with AMG-510 at a concentration of 10 nM and BI-3406 at a concentration of 100 nM either singly or in combination to Binimetinib used at a concentration of 10 nM in the presence (IR-2Gy) and absence of irradiation (IR-0Gy). The GTP bound form of RAS was pulled down with His-tagged fusion protein corresponding to RAS binding domain of Raf-1 conjugated to agarose beads. The RAS-GTP bound to the beads, and the total RAS levels were identified using anti-RAS antibodies. GAPDH was used as a loading control (**a**,**c**). Comparative densitometric analysis of normalized RAS-GTP between the non-irradiated and irradiated groups has been shown as means ± SD for both cell lines in the bar diagrams on the right (**b**,**d**), and statistical significance was stated accordingly (* *p* < 0.05, ** *p* < 0.01, *** *p* < 0.001, ns = non-significant).

To determine the ability of KRAS (AMG-510 and BI-3406) and MEK (Binimetinib) inhibitors to inhibit RAS-GTP complex formation, isoform-specific RAS-GTP binding (RBD) pull-down assay was performed in the absence and presence of irradiation, and the expression level of the active GTP bound state of RAS was measured in both the KRAS mutated cell lines (Figure 6). Interestingly, we observed that either of the KRAS inhibitors, irrespective of being G12C mutation specific (AMG-510) or pan-KRAS (BI-3406), when used as single agent treatment significantly suppressed RAS-GTP levels at low concentrations of 10 and 100 nM, respectively, as compared to controls in KRAS G12C mutant MiaPaCa cells, whereas the MEK inhibitor Binimetinib did not show any tangible reduction of GTP bound RAS. Concomitant irradiation caused slightly further reduction of RAS-GTP upon AMG510 plus Binimetinib treatment (Figure 6a,b). This additive downregulation of RAS activation conferred by concomitant irradiation along with combined KRAS plus MEK inhibition was more pronounced for the KRAS inhibitor BI-3406 in MiaPaCa cells (Figure 6d).

Of note and in line with previous observations on cell growth, clonogenicity, and anchorage-independent growth as described above, AMG510 treatment had no effect on active RAS (RAS-GTP) levels in G12D mutant Panc-1 cells. BI-3406 monotherapy was also not shown to down-regulate RAS-GTP levels in Panc-1 cells, while combined KRAS and MEK inhibition by means of BI-3406 plus Binimetinib combination treatment lead to significant reduction of RAS-GTP levels (Figure 6c,d).

**Figure 7:**
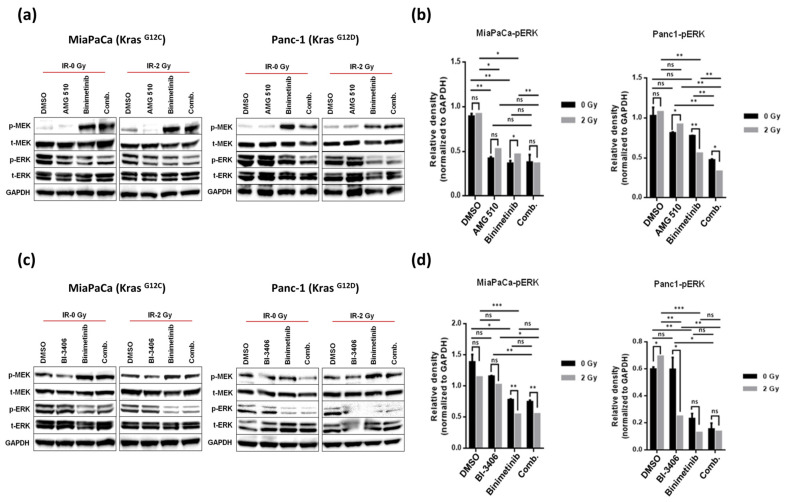
Immunoblots of pMEK and pERK1/2 in both KRAS mutant cell lines, namely MiaPaCa**^KrasG12C^** and Panc-1**^KrasG12D^** (**a**,**c**). Both the cell lines were treated with AMG-510 at a concentration of 10 nM and BI-3406 at a concentration of 100 nM singly or in combination with Binimetinib used at a concentration of 10 nM under low-dose irradiation (IR-2 Gy) or no irradiation (IR- 0Gy). GAPDH was used as a loading control. Comparative densitometric analysis for normalized pERK1/2 between the non-irradiated and irradiated groups has been shown as means ± SD for both cell lines in the bar diagrams on the right (**b**,**d**), and statistical significance was stated accordingly (* *p* < 0.05, ** *p* < 0.01, *** *p* < 0.001, ns = non-significant).

Signaling via the MAPK pathway in response to mono, dual, or triple therapy consisting of small-molecule KRAS or MEK inhibitors, respectively, with or without concomitant irradiation was evaluated via immunoblotting for phosphorylated-MEK (pMEK) and its downstream targets, phosphorylated-ERK1/2 (pERK1/2). As shown in Figure 7a,b, in KRAS G12C mutant MiaPaCa cells, both AMG-510 and Binimetinib were found to be highly potent pathway inhibitors, irrespective of whether they were used as monotherapy or in combination. Both inhibitors demonstrated robust and near-complete abrogation of downstream pERK levels without altering the total levels of ERK, even at a concentration as low as 10 nM. Interestingly, an inverse relationship was noticed between pMEK and pERK levels in response to Binimetinib treatment for both the mutant pancreatic cancer cell lines tested, MiaPaCa and Panc-1 (Figure 7a–d). Our observation that Binimetinib, while still inhibitory, induced high levels of pMEK was in line with the previous reports by others and suggests that MAPK activation might inflict feedback inhibition onto itself, and thus suppression of MAPK signaling by Binimetinib might relieve this inhibitory feedback [42].

Moreover, the addition of irradiation to AMG510 or Binimetinib monotherapy or combined inhibitor treatment, respectively, did not induce additional reduction of pERK levels in MiaPaCa cells (Figure 7b). In Panc-1 cells, AMG510 expectedly did not show any effect on pMEK and pERK expression levels, which also remained unchanged with further addition of irradiation, whereas Binimetinib when used in double or triple combination treatment regimens with either AMG510, irradiation, or both showed significant reduction in the pERK levels, thereby indicating therapeutic synergism (Figure 7a,b).

On the other hand, the pan KRAS inhibitor BI-3406 applied as monotherapy did not alter downstream pERK levels in both cell lines evaluated here but showed marked reduction in pERK levels when combined with irradiation in KRAS G12D mutant Panc-1 cells, thereby hinting at potential radiosensitization conferred by BI-3406. Also, BI-3406 when combined with Binimetinib showed significant downregulation of pERK levels, and this effect was significantly enhanced upon addition of irradiation (Figure 7c,d).

### 2.5. KRAS/MEK Inhibition Sensitizes KRAS Mutant Pancreatic Cancer Cells to Therapeutic Irradiation and Induces Apoptosis

**Figure 8:**
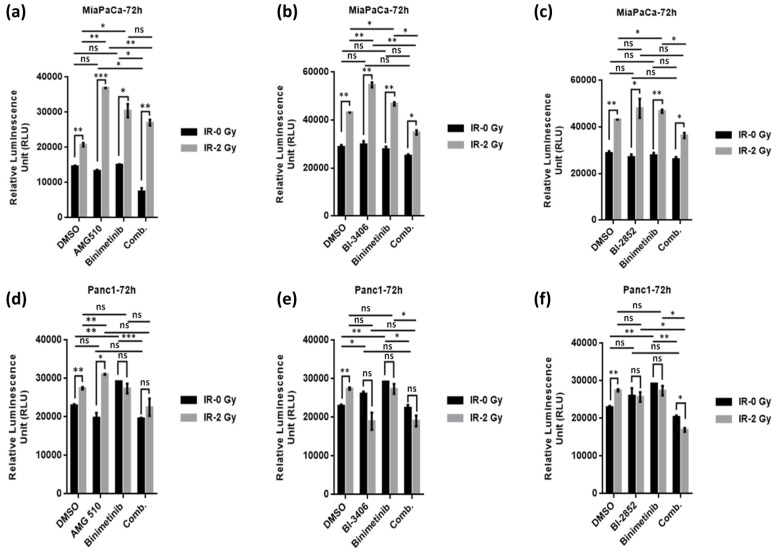
Apoptosis induction by measuring the Annexin V binding (luminescence) from KRAS mutated cell lines, MiaPaCa**^KrasG12C^**, and Panc-1**^KrasG12D^**, respectively, after exposing them to AMG-510 at a concentration of 10 nM (**a**,**d**), BI-3406 at a concentration of 100 nM (**b**,**e**), and BI-2852 at a concentration of 5 µM (**c**,**f**) singly or in combination with Binimetinib at a concentration of 10 nM in the absence (IR-0Gy) and presence of irradiation (IR-2Gy). The results are shown as means ± SD, and the level of significance has been indicated accordingly (* *p* < 0.05, ** *p* < 0.01, *** *p* < 0.001, ns = non-significant).

Induction of apoptosis represents one of the major mechanisms of cell death following exposure to irradiation; therefore, we studied the effect of irradiation (2 Gy) alone or in combination with KRAS and MEK inhibitors, namely AMG510, BI-3406, and Binimetinib, administered either as monotherapy or in combinatorial regimens using RealTime-Glo Annexin V Apoptosis assays (Figure 8). Of interest, irradiation alone at 2 Gy significantly induced apoptosis in both KRAS mutant cell lines examined here. However, combined irradiation plus KRAS/MEK inhibition (by means of AMG510, BI-3406, BI-2852, and Binimetinib, respectively) markedly enhanced induction of apoptosis (Figure 8a–c). Of note, this pronounced combinatorial effect was seen only in KRAS G12C mutant MiaPaCa but not in KRAS G12D mutant Panc-1 cells (Figure 8d–f).

## 3. Discussion

Pancreatic cancer carries an exceptionally poor overall prognosis and shows relative insensitivity towards many antineoplastic agents, including classical cytotoxic chemotherapeutic drugs as well as currently available immunotherapies [43,44,45,46]. Hence, therapeutic options for the majority of advanced disease stages are limited, and novel therapeutic approaches are urgently required [47].

Pancreatic cancer is driven by oncogenic KRAS variants in more than 90% of cases [29], and even those cases with wild type KRAS often show alternative alterations that activate oncogenic signaling through the RAS/MAPK pathway [48]. Therefore, oncogenic KRAS signaling has long been identified as a major driver underlying pancreatic carcinogenesis and has been suggested as putative target for therapeutic intervention [49]. Moreover, functional genomic annotation studies found a strong association of KRAS mutations with cellular KRAS dependency, underlining the assumption that KRAS mutations act as main oncogenic drivers in a majority of cases [50]. However, the development of suitable drug candidates has proved to be challenging. There is a notable variation in oncogenic KRAS allelic distribution across different tumor types; while G12C is more prevalent in KRAS mutant adenocarcinomas of the lung (13.6%), G12D, G12V, and G12R represent the most common oncogenic KRAS variants detected in pancreatic cancers (37.0%, 28.2%, and 12.7%, respectively), while G12C is found in only 1.1% of cases [51].

While molecularly targeting oncogenic KRAS-driven malignancies has long been deemed impossible, the introduction of Sotorasib (AMG-510) as first-in-class small molecule KRAS G12C inhibitor has dramatically changed this paradigm and in fact now represents a standard therapeutic option for KRAS G12C mutant NSCLC patients [52]. But despite showing promise, these compounds are also plagued with the limitation of being effective only in the subset of KRAS G12C specific mutant tumors. Unlike in NSCLC, G12C mutations represent only a minor fraction of KRAS mutations detected in PDAC, yet this therapeutic approach has been evaluated in gastrointestinal carcinomas including pancreatic cancer, albeit with limited success. In a phase 2 CodeBreaK100 trial, Sotorasib monotherapy showed only modest clinical activity in KRAS G12C mutant colorectal cancer with an objective response in only 6/62 (9.7%) cases [53]. Likewise, in the KRYSTAL-1 trial, another G12C specific KRAS inhibitor, Adagrasib (MRTX849), showed objective response in 33% of cases when given as monotherapy [54]. Inhibitors specifically targeting other oncogenic KRAS alleles are currently under active preclinical and early clinical evaluation, including the G12D inhibitors MRTX1133 and BI-KRAS-G12D1-3 that showed promising on-target activity in preclinical models of gastrointestinal tumors including pancreatic cancer [51,55].

Also, in recent years, various groups of compounds entered the arena of preclinical evaluation that are designated as indirect pan-KRAS inhibitors, such as SHP2 or GEF-SOS1 inhibitors among others [51]. Another line of development aims to generate drug candidates with the ability to act as direct pan-KRAS inhibitors by inhibiting various different oncogenic KRAS alleles, such as a compound designated as BI-panKRAS3 [51].

Nevertheless, in all these studies so far, response rates and duration of response seem to be limited in pancreatic cancer, possibly due to emergence of secondary resistance-mediating oncogenic variants or due to clonal selection of pre-existing alternative oncogenic driver KRAS alterations [29]. Therefore, identification of co-vulnerabilities and development of suitable combination therapy approaches to achieve long-lasting remissions in the majority of patients and to overcome adaptive resistance is currently an area of active research [56]. Increasing evidence suggests that identification of such combinatorial strategies aiming at synthetic lethality with oncogenic KRAS inhibition might be highly tissue-specific and vary with individual sites of tumor origin and with individual patterns of accompanying oncogenic genomic variants [57]. In KRAS G12C mutant colorectal cancer, combinatorial KRAS inhibition with Sotorasib and EGFR blockade by means of Panitumumab was found to be superior to either monotherapy in the phase 3 CodeBreaK300 trial, and similarly Adagrasib in combination with Cetuximab showed enhanced efficacy with considerably higher response rates as compared to monotherapy [58]. MEK and PI3K inhibition have previously been found to synergize and inhibit oncogenic KRAS signaling [59]. More recent evidence strongly suggests therapeutic synergism of KRAS inhibition in combination with immune checkpoint blockades in pancreatic cancer [60].

The role of radiation therapy in pancreatic cancer is still a matter of ongoing research [61,62], and particularly combination regimens with potential radiosensitizers are actively searched for.

The current work presented here applies a structured approach to screen for potential therapeutic synergisms of direct KRAS inhibition by means of three different compounds, AMG510, BI-3406, or BI-2852, respectively, with concomitant MEK inhibition using Binimetinib and radiation therapy in pancreatic cancer cells with different KRAS status. While MiaPaCa and Panc-1 cells are driven by two different oncogenic KRAS variants, G12C and G12D, respectively, BxPC-3 cells are KRAS wild-type.

Due to the high complexity and vast number of potential combinatorial therapy regimens, it seems crucial to systematically and thoroughly screen such combination regimens using suitable in vitro model systems in order to identify promising combinations that might subsequently be further pursued for in vivo evaluation.

Of note and in line with previous observations, both Sotorasib and BI-3406 showed robust on-target efficacy when applied as monotherapy in various assays using KRAS G12C mutant MiaPaCa pancreatic cancer cells that was further enhanced by concomitant MEK inhibition with Binimetinib, while efficacy of BI-2852 was limited. It is a novel observation in the study presented here that these KRAS plus MEK inhibitor combination regimens apparently show marked therapeutic synergism with concomitant irradiation across several of the in vitro assays presented here, suggesting this might pose a previously not recognized and therefore understudied scope for therapeutic synergism [58,62].

From a clinical point of view, there is a dire need to target those oncogenic KRAS variants that are more prevalent in pancreatic cancer, including KRAS G12D. Therefore, it is an important observation of this current work that in the model systems examined here, BI-3406 apparently had good on-target in vitro efficacy in KRAS G12D mutant Panc-1 cells, which was again most pronounced when combined with MEK inhibition with Binimetinib plus concomitant radiotherapy. Since therapeutic KRAS inhibition has recently been suggested to sensitize pancreatic cancer cells to immune checkpoint inhibitor therapy and clinical evidence from other tumor entities such as malignant melanoma or non-small cell lung cancer point at potential further sensitization to immunotherapy by concomitant irradiation; one might even envision a quadruple approach that also includes immune checkpoint inhibitors in these regimens [60]. This approach appears even more possible, since toxicity profiles are distinct from each other with usually little overlap [63].

Accumulating recent evidence suggests that specific oncogenic KRAS variants show distinct biological relevance and may predict outcome and response to therapy in pancreatic cancer [64]. It is therefore an important task for future work to include other, potentially more rare oncogenic KRAS variants at different positions of the KRAS gene as well as their context-specific behavior in combination with other oncogenic driver variants in other genes and response to other molecularly targeted inhibitor combinations [65]. This represents an ongoing major challenge of translational cancer research and could be addressed by incorporating data from multicenter networks on personalized genomic therapies currently established [66].

Recent evidence suggests that three-dimensional organoid models may be superior to classical two-dimensional cell culture assays in predicting in vivo efficacy in human patients [67,68]. It is therefore a very promising finding of this study that therapeutic efficacy as discussed above could also be validated using a three-dimensional cancer cell colony growth soft agar assay.

Taken together, the preclinical evidence presented here hints at a potential therapeutic synergism of KRAS inhibition by means of AMG510 (Sotorasib) or BI-3406 in combination with MEK inhibition by Binimetinib and concomitant irradiation in KRAS mutant pancreatic cancer. This combinatorial approach should therefore be further evaluated using suitable in vivo model systems in the next step forward.

## 4. Materials and Methods

### 4.1. Cell lines and Drugs

Human pancreatic cancer cell lines MiaPaca**^KrasG12C^**, Panc-1**^KrasG12D^**, and BxPC-3**^KrasWT^** cells with varying mutational status of KRAS were cultured in DMEM media (Life Technologies, Darmstadt, Germany) supplemented with 10% FBS, 1x penicillin/streptomycin (Life Technologies, Darmstadt, Germany) and 5 μg/mL plasmocin (InvivoGen, San Diego, CA, USA). All the cell lines were grown in a humidified atmosphere at 37 °C in the presence of 5% CO_2_ and were routinely tested for mycoplasma infection using a PCR-based assay as described elsewhere [69]. AMG-510 (Sotorasib KRAS G12C inhibitor) and Binimetinib (MEK inhibitor) were purchased from MedChemExpress, NJ, USA. BI-3406 and BI-2852 were kindly provided by Boehringer Ingelheim. BI-3406 is a highly potent and selective Son of Sevenless 1 (SOS1):: Kirsten rat sarcoma viral oncogene (KRAS) protein interaction inhibitor. This small molecule inhibitor binds to the catalytic site of SOS1, inhibiting the interaction with RAS-GDP. This significantly reduces formation of GTP-loaded KRAS (activated KRAS), thereby inhibiting downstream MAPK signaling [70]. The compound BI-2852 is a potent inhibitor of the KRAS switch I/II pocket. It directly targets and inhibits the active GTP-bound form of KRAS. It modulates pERK, and its antiproliferative effects were observed in KRAS mutant NCI-H358 cells [71,72]. Stock solutions of each drug (20 mM) were prepared in DMSO, and aliquots were stored at −20 °C for further use. The compounds were diluted in culture media at their respective concentrations prior to each experiment. Combinatorial regimens were applied to identify potential additive or synergistic therapeutic efficacy [73].

### 4.2. Irradiation

Irradiation was performed at room temperature using a Precision Multirad 225 X-ray system (Wheeling, IL, USA) operated at 225 kV and 17.8 mA, at a dose rate of 6 Gy/min. Stock solutions of AMG-510, BI-3406, BI-2852, and Binimetinib dissolved in DMSO were further diluted in cell culture media to make respective concentrations. Drugs were added to the plated cells 1 h prior to irradiation exposure and maintained for the time duration required for the respective experiment. The applied radiation dose used was 2 Gy and compared with no irradiation (0 Gy) for the respective experiments.

### 4.3. Cell Viability Assay

Cell viability was determined using Cell Titer Glo 2.0 luminescence assay (Promega, Madison, WI, USA), and the experiment was conducted in accordance with the instructions provided by the manufacturer. Briefly, 5000 cells/well were plated in full growth media and were treated with AMG-510, BI3406, BI-2852, or Binimetinib either singly or in combination for 1 h. Post one hour, cells were irradiated at 0 or 2 Gy and further incubated for 72 h, at which time point the assay was terminated by adding 50 µL of CellTiter-Glo^®^ 2.0 Assay reagent (Promega, Madison, WI, USA). The contents were mixed for 2 min at 300rpm on an orbital shaker, and the plates were incubated for 10 min at room temperature to stabilize luminescent signals. Luminescence was recorded using a Fluoroskan FL Microplate Luminometer (Fisher Scientific GmBH, Schwerte, Germany) where the luminescent signal generated was proportional to the amount of ATP present in viable cells. The growth inhibition was thus determined and compared by measuring the cellular ATP levels between the non-radiated and irradiated groups. All experiments were set up in triplicates, and the results were plotted as means ± SD.

### 4.4. Clonogenicity (2D) Assays

MiaPaca**^KrasG12C^**, Panc-1**^KrasG12D^**, and BxPC-3**^KrasWT^** cells were seeded in a six-well plate at a concentration of 1000 cells/mL and treated with AMG-510, BI-3406, and BI-2852 either singly or in combination with the MEK inhibitor Binimetinib. Post 1 h of drug addition, cells were exposed to 2 Gy irradiation and maintained for the required period of the experiment. Once the colony clusters became visible, cells were fixed using 70% ethanol followed by staining with 0.05% (*w*/*v*) crystal violet (Sigma Aldrich, Steinheim, Germany) and visualization by trans-UV illumination (BioRad, Hercules, CA, USA). The stained colonies were counted, and the colony counts were expressed as mean colony count ± SD.

### 4.5. Anchorage Independent (3D) Soft Agar Clonogenic Assays

Colony formation in 3D soft agar matrix was assessed for AMG-510, BI-3406 and BI-2852 either singly or in combination with Binimetinib in the absence and presence of irradiation. The assay was set up in a six-well plate as described previously [74,75]. Briefly, a base agar layer was formed by mixing 2 mL of media and 1% agarose on top of which a second layer of 2 mL of media containing 0.7% agarose, 10,000 cells, and the respective desired drug concentrations (singly or in combination) was poured and allowed to solidify. Finally, 2 mL of media containing the respective drugs was added on top of the agarose layers. Post 1 h of drug addition, plates were exposed to 2 Gy irradiation or no irradiation. Subsequently, the plates were kept in a tissue culture incubator maintained at 37 °C and 5% CO_2_ for 3–4 weeks to allow for colony growth. When the colonies became visible, the assay was terminated, and the colonies were stained and counted. All assays were performed in triplicates, and the colony counts were expressed as mean colony count ± SD.

### 4.6. Western Blots

MiaPaca**^KrasG12C^**, Panc-1**^KrasG12D^**, and BxPC-3**^KrasWT^** cells were treated with desired concentrations of all respective KRAS inhibitors either singly or in combination with the MEK inhibitor in the presence or absence of irradiation for 72 h. Next, cells were lysed using radioimmunoprecipitation assay buffer (1% Igepal CA630, 0.5% sodium deoxycholate, 0.1% SDS, 2 mM EDTA) supplemented with protease and phosphatase inhibitor cocktails (Sigma-Aldrich, Steinheim, Germany). An amount of 50 µg of total protein was separated using 4% to 12% Nupage bis-tris gels (Life Technologies, Darmstadt, Germany) and transferred onto PVDF membranes (Millipore, Billerica, MA, USA). The blots were blocked using either 5% (*w*/*v*) BSA or 5% (*w*/*v*) milk in TBST for 1 h and then later probed using primary antibodies against phospho-ERK, ERK, phospho MEK, MEK, cleaved Caspase-3, p27 (all used at a dilution of 1:1000; Cell Signaling, Danvers, MA, USA), PARP, and GAPDH (1:10,000; Proteintech Germany GmBH, Munich, Germany) as well as HRP-coupled secondary antibodies directed against rabbit or mouse IgG, respectively (1:2000, Cell Signaling). Detection was performed as previously described [76]. Quantification was performed by densitometry using ImageJ analysis software (https://imagej.nih.gov/ij/, accessed on 16 January 2023).

### 4.7. RealTime-Glo^TM^ Annexin V Apoptosis Assay

Apoptosis was determined using a RealTime-Glo Annexin V Apoptosis kit (Promega, Madison, WI, USA), and the experiment was performed in accordance with the recommendations provided by the manufacturer. Briefly, 5000 cells/well (MiaPaca**^KrasG12C^**, Panc-1**^KrasG12D^**) were seeded in a 96-well flat-bottomed white plate and treated with KRAS and MEK inhibitors in the presence or absence of irradiation. The cells were then incubated for 72 h, the time point at which the assay was terminated. To terminate the experiment, 100 µL of 2x detection reagent (each time made fresh according to the manufacturer’s protocol) was added to each well, mixed on a plate shaker for roughly 60 s at 300 rpm, and luminescence was recorded using a Fluoroskan FL Microplate Luminometer (Fisher Scientific GmBH, Schwerte, Germany).

### 4.8. Active RAS Pull-Down Assay

Levels of active GTP-loaded RAS were determined using a RAS pull-down Activation assay Biochem Kit (Cytoskeleton, Denver, CO, USA; # BK008) after treating MiaPaCa**^KrasG12C^** and Panc-1**^KrasG12D^** cells with the respective desired concentrations of inhibitors (singly or in combination) in the presence of low-dose radiation of 2 Gy or no irradiation. Briefly, 400 µg of cell lysates collected in lysis buffer were mixed with GST-Raf1-RBD protein bound colored glutathione agarose beads and incubated at 4 °C on a rotator for 1 h. Bound proteins washed and eluted with SDS sample buffer were then subjected to SDS-Page gel electrophoresis. After running and transfer of protein samples, membranes were probed with RAS specific primary antibody followed by incubation with HRP-coupled secondary antibody and detection for GTP bound form of active RAS protein. Quantification was performed by densitometry using ImageJ analysis software (https://imagej.nih.gov/ij/ accessed on 10 January 2023).

### 4.9. Statistical Analysis

Two-tailed Student’s *t*-test, Mann–Whitney U-test, and Kruskal–Wallis analysis were performed using Graph Pad Prism for Windows version 6. Unless indicated otherwise, results were expressed as mean ± SD. *p* < 0.05 was set as the threshold of statistical significance.

## Data Availability

All experimental data used to support the findings of this study are included within the article.

## Supplementary Materials

The following supporting information can be downloaded at: https://www.mdpi.com/article/10.3390/ijms25116249/s1.

## Abbreviations

AMG-510	Sotorasib -KRAS G12C inhibitor
BI-2852	KRAS switch I/II pocket inhibitor
BI-3406	SOS1::KRAS inhibitor
Binimetinib	MEK inhibitor
BRAF	Murine Sarcoma Viral (V-Raf) Oncogene Homolog B1
EGFR	Epidermal Growth Factor Receptor
ERK	Extracellular -signal- Regulated Kinase
KRAS	Kirsten Rat Sarcoma viral oncogene
MAPK (MEK)	Mitogen -Activated Protein Kinase
PI3K	Phosphatidylinositol 3-kinase
RAF	Rapidly Accelerated Fibrosarcoma
RAF-MAPK	RAF-mitogen activated protein kinase
RAL	Ras Linked Protein
RALGDS	RAL Guanine nucleotide Dissociation Stimulator
RAS	Rat Sarcoma
SOS1	Son Of Sevenless homolog 1
