# Supplementary material for: Triple Blockade of Oncogenic RAS Signaling Using KRAS and MEK Inhibitors in Combination with Irradiation in Pancreatic Cancer
Source: Int J Mol Sci. 2024 Jun 6;25(11):6249. doi: 10.3390/ijms25116249 (PMC11172716; doi:10.3390/ijms25116249)
Supplement: Supplementary file 1 [file ijms-25-06249-s001.zip › ijms-3008149-supplementary.pdf]

Fig. S1: Supplementary Figure

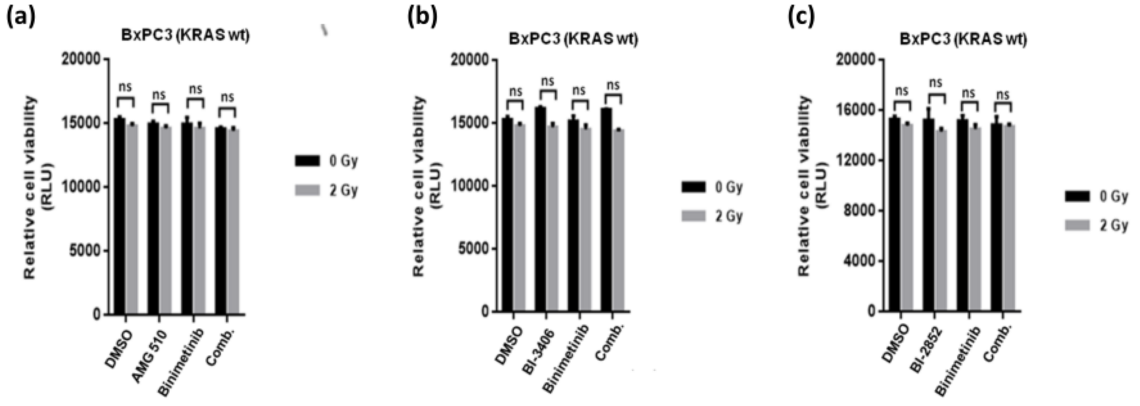

Figure S1: Relative cell viability of BxPC3 ^{KrasWT} cells treated with AMG-510, BI-3406, BI-2852 at a concentration of 10 nM, 100 nM, 1 μM respectively, either singly or in combination with Binimetinib used at a concentration of 10 nM exposed to either low-dose irradiation (2 Gy) or no irradiation (0 Gy). The mitochondrial metabolic function (viability) is plotted as relative luminescence unit (RLU) of treated cells in relation to mock treated cells. Results of three independent experiments are shown and are expressed as means ± SD. Statistical significance has been indicated as * $p < 0.05$, ** $p < 0.01$, *** $p < 0.001$, ns= non-significant.
